# Predictors and outcomes in primary depression care (POKAL) – a research training group develops an innovative approach to collaborative care

**DOI:** 10.1186/s12875-022-01913-6

**Published:** 2022-12-02

**Authors:** J Gensichen, K Lukaschek, C Jung-Sievers, P Falkai, A Schmitt, P Henningsen, T Dreischulte, G Pitschel-Walz, H Krcmar, M Böhm, B Prommegger, K Linde, A Drescher, P Schönweger, C Haas, C Brand, P Younesi, J Vukas, V Brisnik, H Schillok, J Raub, L Kaupe, K Biersack, F Gökce, J Eder, L Hattenkofer, Ph Reindl-Spanner, V von Schrottenberg, C Teusen, Ph Sterner, M Bühner, A Schneider, for the POKAL group, Hannah Schillock, Regina Wehrstedt von Nessen-Lapp, Kirsten Lochbühler

**Affiliations:** 1 Institute of General Practice and Family Medicine, University Hospital, LMU Munich, Nussbaumstraße 5, 80336 Munich, Germany; 2 Chair of Public Health and Health Services Research, Institute for Medical Information Processing, Biometry and Epidemiology, Pettenkofer School of Public Health, LMU Munich, Munich, Germany; 3 Department of Psychiatry and Psychotherapy, University Hospital, LMU Munich, Munich, Germany; 4 Laboratory of Neurosciences (LIM-27), Institute of Psychiatry, University of São Paulo (USP), São Paulo, Brazil; 5 Department of Psychosomatic Medicine and Psychotherapy, Klinikum Rechts Der Isar, Technical University of Munich, Munich, Germany; 6 Institute of General Practice and Health Services Research, School of Medicine, Technical University of Munich, Munich, Germany; 7 School of Computation, Information and Technology, Technical University of Munich, Garching, Munich, Germany; 8 Department of Informatics, University of Applied Sciences Landshut, Landshut, Germany; 9 Department of Psychology, Psychological Methods and Assessment, Ludwig Maximilian University of Munich, Munich, Germany

**Keywords:** Primary care, Depression, Multimorbidity, Research training group, Chronic care model (CCM)

## Abstract

**Background:**

The interdisciplinary research training group (POKAL) aims to improve care for patients with depression and multimorbidity in primary care.

POKAL includes nine projects within the framework of the Chronic Care Model (CCM). In addition, POKAL will train young (mental) health professionals in research competences within primary care settings. POKAL will address specific challenges in diagnosis (reliability of diagnosis, ignoring suicidal risks), in treatment (insufficient patient involvement, highly fragmented care and inappropriate long-time anti-depressive medication) and in implementation of innovations (insufficient guideline adherence, use of irrelevant patient outcomes, ignoring relevant context factors) in primary depression care.

**Methods:**

In 2021 POKAL started with a first group of 16 trainees in general practice (GPs), pharmacy, psychology, public health, informatics, etc. The program is scheduled for at least 6 years, so a second group of trainees starting in 2024 will also have three years of research-time. Experienced principal investigators (PIs) supervise all trainees in their specific projects. All projects refer to the CCM and focus on the diagnostic, therapeutic, and implementation challenges.

**Results:**

The first cohort of the POKAL research training group will develop and test new depression-specific diagnostics (hermeneutical strategies, predicting models, screening for suicidal ideation), treatment (primary-care based psycho-education, modulating factors in depression monitoring, strategies of de-prescribing) and implementation in primary care (guideline implementation, use of patient-assessed data, identification of relevant context factors). Based on those results the second cohort of trainees and their PIs will run two major trials to proof innovations in primary care-based a) diagnostics and b) treatment for depression.

**Conclusion:**

The research and training programme POKAL aims to provide appropriate approaches for depression diagnosis and treatment in primary care.

## Background

Population aging produces an increase in the prevalence and complexity of mental illnesses. At the same time, crises such as the current pandemic and/or climate-associated challenges cause mental health topics to gain in relevance, and increase the burden on health care systems [1–3]. Depression is a common and heterogeneous condition with a chronic and recurrent natural course, and it is frequently treated in the primary care setting [4]. As general practitioners (GPs) usually make the initial diagnosis and, particularly in the case of mild and moderate disorders, are responsible for treating patients with depression, they play an important role in their care [4]. However, recognizing, treating and managing depression in a primary care setting can be challenging, particularly when patients have multiple comorbidities [5]. The challenges associated with early recognition and treatment of depression in primary care have been widely documented and include a combination of patient-, provider-, and system-related barriers [4, 6]. In short, somatic comorbidities can mask a depression, making the selection of a suitable therapy and the decision how to implement it more difficult. This may compromise both the success of the therapy and the safety of the depression treatment. Guideline recommendations can only be implemented to a limited extent in patients with mental illness or multimorbidity [7–9]. Mental health disorders are more prevalent in primary care patients with multimorbidity [9–12].

The research training group on predictors and outcomes in primary depression care (POKAL, PrädiktOren und Klinische Ergebnisse bei depressiven ErkrAnkungen in der hausärztLichen Versorgung) aims to improve approaches for depression diagnosis and treatment in primary care. In addition POKAL will train young (mental) health professionals in research competences within primary care settings. POKAL focuses on the chronic course of depression among patients with multimorbidity, using the definition of multimorbidity established in the German MultiCare study (> 2 chronic diseases) [13]. Depression is more common in patients with chronic somatic diseases [14], and, as well as occurring independently of a physical illness, can also have both somatic causes and consequences [4].

GPs treating patients with depression and/or multimorbidity face the following challenges:

- *Diagnostic challenges—*GPs are confronted with complex diagnostic clusters that are often difficult to break down into separate parts due to "disease-disease" interactions [5, 6, 15, 16]. Distinguishing between the symptoms of depression and somatic or non-specific, functional or somatoform, physical complaints (including those caused or worsened by adverse drug reactions) is particularly challenging. Furthermore, it may lead to increased consultation of medical staff and to iatrogenic harm, e.g. due to polypharmacy [17, 18]. Although often confronted with a high workload and little time, GPs must nonetheless make reliable diagnoses [19]. Signalling symptoms (“red flags”) can help to structure diagnostic procedures through the use of “opportunistic screening”, followed by, for example, use of a self-rating instrument and an ICD-10-based diagnostic checklist [20].
- Multimorbidity is one important factor in increased risk of suicidal ideation [21]. A recent meta-analysis including a total of 19 studies found that the pooled odd ratio (OR) for the association between multimorbidity and suicidal ideation was 2.90 (95%CI 2.29–3.67, *P* < 0.001)[22]. GPs should assess suicidal ideation among patients with multimorbidity, particularly among patients with multimorbid physical and mental health conditions, without endangering the important trusting relationship between the patient and the GP [23].
- *Challenges of treatment*—Depression modulates disease trajectories, even in the chronic somatic illnesses that are usually treated by general practitioners [24]. Drug interactions ("drug-drug", "drug-disease") change the prognosis of therapy regimens and make it more difficult for general practitioners to monitor the course of treatments. For patients and their primary care physicians, the treatment of chronic psychiatric and chronic somatic illnesses is highly fragmented due to the involvement of a large number of different health care professionals [25]. Further problems are insufficient patient involvement participation [26–28] and inappropriate long-time anti-depressive medication [29, 30]. Especially in the context of multimorbidity in primary care, patient participation and coordinated long-term concepts are necessary in order to counteract existing uncoordinated and fragmented care [31, 32].
- *Challenges of implementation* – Instruments aimed at promoting the implementation of current research findings relating to depression in GP practice, such as evidence-based guidelines, are limited in their effectiveness because of individual factors such as knowledge and attitude [33, 34], previous experience with adverse drug reactions, and treatment preferences [35]. Their effectiveness is also held back by contextual factors such as costs, finding the necessary time, making use of supportive community resources and improving access to care [36]. The implementation of innovations improves when applied in the shape of coordinated strategies [37]. Thus, "nudging" (gentle steering, pushing) can influence medical decision heuristics concerning guideline adherence. Digital applications are being increasingly used and, provided they are user friendly, also have the potential to improve therapy and help monitor depression treatment [38]. However, the involvement of patients with depression in the development and use of such applications is a challenge, given the lack of motivation often associated with depression.

This paper provides an overview of the background, concept, aims, and projects of a German research training school on mental health in primary care (POKAL).

## Methods

### POKAL’s guiding model: the chronic care model

**Fig. 1:**
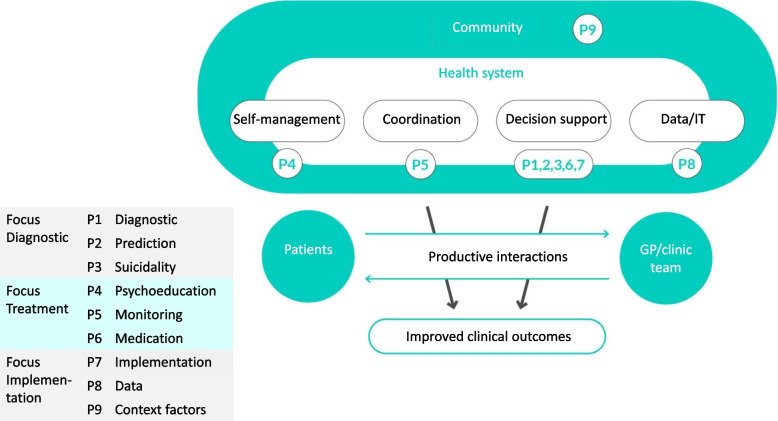
POKAL projects (P1-P9) within the CCM ©POKAL 2022, adapted from ACP-ASIM Journals and Books. www.act-center.org

Various theoretical models exist on the treatment of multimorbidity, which are based on the *collaborative care* of patients with multiple chronic illnesses, including mental illnesses [39–41]. One of those collaborative care models, the chronic care model (CCM) is the guiding model for POKAL. The CCM, an evidence-based approach to structuring clinical care and to ensure care is coordinated, patient-centred, and anticipatory [42, 43]. The CCM operationalizes principles of collaborative care, with the ultimate aim of improving access to evidence-based mental health therapy for patients in a primary care setting [44, 45]. Studies have shown that when clinical practices are designed according to the CCM, quality and outcomes of care improve for various chronic conditions [39, 42, 46]. The CCM is based on the assumption that improved clinical outcomes result from a "productive interaction" between treating physicians and their patients. The combination of four principles promotes this interaction (see Fig. 1): self-care (self-management, behavioural activation), coordination (teamwork, case management), decision support (evidence-based guidelines), IT/data (clinical patient, practice and routine data)[47].

Patient self-management, or the active involvement of patients in the treatment process, strengthens the patient's role. Among other things, it involves assisting in making treatment decisions, keeping an eye on clinical results, generating personal health data, and dealing with special disease situations safely.

Coordination of all participants, and interdisciplinary task sharing, for example within practice teams, i.e. family doctors, medical assistants and patients, but also between family doctors, patients and psychiatrists / psychotherapists. The aim of coordination is to improve clinical care processes and treatment, e.g. through monitoring (case management).

Decision-making support Clinical diagnostic and therapeutic decisions are science-based, and take into account, for example, evidence-based guidelines for physicians and patients.

IT and data collection ensure quality assurance in clinical care. Patient records and registers, as well as routine and patient-generated health data, are used to plan the treatment of individual patients and entire patient groups, e.g. in family practices.

These principles are embedded in and influenced by the community and health care system: In order to promote awareness and mobilize resources, close links with wider community networks and agencies, e.g. self-help groups and voluntary organizations, are essential [48]. The health care system provides the systemic infrastructure that is required to sustain planned care.

Finally, the CCM emphasizes the need to engage patients as active partners in the management of their condition(s), as they need to develop new skills and make changes in their lifestyles. GPs help their patients make effective use of self-help strategies [48]. In this respect, establishing an effective patient-GP interaction also results in improved clinical outcomes.

Mental health programmes designed on the basis of the CCM integrate, for example, elements of the patient's own self-management of "stress". To improve coordination, they also use monitoring tools and intensive case management involving telephone contacts and coaching, and include elements from cognitive behavioural therapy, nutritional therapy, exercise therapy and principles of social activation that take advantage of digital and non-digital tools [49, 50].

### Data and outcomes

Evidence shows that collaborative care models such as the CCM can reduce behavioural health disparities in vulnerable subgroups and improve access, quality of care, and health outcomes for behavioural health conditions [51, 52]. Evidence from a meta-analysis has shown that comorbid patients with depression that were treated according to collaborative care principles had higher rates of adherence to antidepressants and other medications [53].

However, effectiveness varies in real-world settings, and probably depends on the provided implementation support [54]. To measure the effect of collaborative care models, clinical outcomes as well as other outcomes relevant to mental health should be considered [55]. Literature has shown evidence that collaborative care across different mental health disorders produces better outcomes than usual care in terms of clinical symptoms, adherence to medication, mortality, quality of life (QOL), and social role functioning [55, 56]. Unützer et al*.* found greater overall QOL at 12-month follow up, compared with usual care [57]. More specifically, evidence exists that collaborative care has a more positive effect than usual care on mental health QOL outcomes in the short-, medium-, and long-term [58]. With regard to social role functioning, a systematic review by Hudson et al. (2016) investigated the positive effect of collaborative care in patients with depression on the degree to which patients returned to normal behaviour in their work and recreational environments, and assumed their usual social roles [59].

In order to enable research questions beyond those targeted by individual POKAL projects to be investigated, the consortium has systematically developed and agreed to collect a core data set (CDS) from all research participants. Apart from basic socio-demographic variables (age, sex, nationality, marital status, education, employment), previous history of depression, and current medication, the CDS will include validated instruments to assess major mental health disorders (depression: Patient Health Questionnaire (PHQ)-9 [60], anxiety: Generalised anxiety disorder scale (GAD)-7 [61], somatization: PHQ-15 [62], post-traumatic stress disorder (PTSD): PC-PTSD-5 [63]) as well as the Patient Assessment of Chronic Illness Care (PACIC). The PACIC is a self-report tool for patients and measures the extent to which patients report having received treatment in line with various aspects of the CCM. It uses five subscales (Patient Activation/Involvement, Delivery System Design/Decision Support, Goal Setting/Tailoring, Problem Solving/Contextual, Follow-up/Coordination)[64]. Higher scores are indicative of perceptions of better care and can thus be viewed as surrogate measures of patient satisfaction. The original PACIC questionnaire was developed in English and contained 20-items [64]. Each item is scored on a 5-point scale, ranging from 1 to 5, and the overall PACIC is calculated by averaging scores across all 20 items. Two other versions have been validated: a 26-item version called PACIC-5As [65] and an 11-item short version [66], with an 11-point scale ranging from ‘none (0)’ to ‘always (100)’. The latter has been translated into German [67] und is to be used in POKAL.

The CDS provides a common database that will be shared by all involved projects and will enable relationships between socio-demographics, somatic and mental health conditions to be investigated, at the same time as providing flexibility for linkage with other mental health cohorts. In addition to the CDS, all data collection and storage will be standardized and quality assured by means of a jointly developed electronic research file (eCRF). The eCRF provides a common data collection and data entry template for all variables (including the CDS) that are relevant to more than one POKAL project. It can also be adapted to include project-specific data items. The entered data will be de-personalized and stored on a secure central server. Data access will be password protected and initially limited (via passwords) to the POKAL project teams and the central data manager (for quality control purposes). It will be possible to extract anonymized data from one or more projects and make it available to other research teams upon request.

## Results of POKAL

### Research

The aim of POKAL is to significantly improve the care of multimorbid patients with depressive disorders in general practice and to qualify the next generation of scientists to work in this field.

The first cohort of the POKAL research training group will develop and test new depression-specific diagnostics (hermeneutical strategies, predicting models, screening for suicidal ideation), treatment (primary-care based psycho-education, modulating factors in depression monitoring, strategies of de-prescribing) and implementation in primary care (guideline implementation, use of patient-assessed data, identification of relevant context factors). Based on those results the second cohort of trainees and their PIs will run two major trials to investigate innovations in a) diagnostics and b) treatment for depression in primary care.

To achieve its aims, POKAL interacts with service users and health professionals, and actively involve patient representatives, the public and providers.

### Training

A comprehensive qualification concept has been developed that gives POKAL-trainees a well-structured scientific education in parallel to their (clinical) training. The program combines and complements clinical expertise in the diagnosis and treatment of psychosocial diseases by taking advantage of the scientific competencies of GPs, psychiatrists/psychosomatic practitioners and psychologists, as well as members of other clinical (e.g. pharmacists) and technical (e.g. computer scientists) professions. Furthermore, the doctoral candidates will develop skills that will be useful in their future professions in academia and research, as well as in patient care (primary care and psychological), IT and management. POKAL trainees will benefit from the excellent clinical and scientific infrastructure in Munich, and it will be possible for them to obtain doctoral degrees in close collaboration with the Munich Medical Research School (MMRS), which is part of the graduate center of the LMU and the TUM Graduate School (TUM-GS). POKAL also embeds an international aspect in the training: the advisory board is composed of national *and* international advisors. Moreover, each POKAL project has an individual international consultant. POKAL trainees are also supposed to do an internship abroad. All POKAL members are committed to promoting equal opportunities, and three of the POKAL’s nine PIs are women (33%). As this proportion is relatively low, we have recruited 10 (62.5%) female researchers for the doctoral positions. By implementing a “Gender Equality Task Force,” the POKAL consortium aims to promote the careers of female scientists and establish a family-friendly workplace.

### The projects

The individual projects of POKAL and its partners will employ and develop the CCM as a working model (Fig. 1). The principal investigators (PIs) and experts come from different scientific backgrounds (e.g. general practice, psychiatry, psychotherapy, public health, epidemiology, pharmacology, informatics, statistics).

#### Diagnostic projects (P1-P3)

Although the PHQ-9 Depression Screener is an instrument that efficiently rules out clinically significant depression, further diagnostic tests do not confirm suspected depression in about 60% of patients that screen positively. In patients with multimorbidity, the risk of false positive screenings is further exacerbated by the strong correlation between somatic diseases and the somatic symptoms of depression (e.g. fatigue, lack of concentration, sleep disorders) [68]. The aim of projects in this priority area is therefore to systematically develop and evaluate new approaches, including the use of general practitioner heuristics, to attain an early and specific diagnosis of depression in patients with somatic comorbidities in primary care settings. In addition to the standard diagnosis described in ICD 10, the first cohort of doctoral students will identify further diagnostically relevant factors. For this purpose, project 1 (short title "Diagnostics") will look at the heuristics used by family doctors and, for example, focus on the psychosocial and socio-economic patient-related aspects used in the identification of depression and when making therapy decisions in patients with multimorbidity. Similarly, project 2 (short title "Prediction") aims to identify physiological factors such as blood-based measures and heart rate variability as a basis for the improved diagnosis of depression. In accordance with the 'Stages in the development of clinical prediction rules' [69], the identified aspects will be combined to develop a new algorithm, which will then be validated both internally and externally. A validated algorithm should then be available by the end of the first funding period. In project 3 (short title "Suicidality") opportunities for suicide prevention in primary care will be investigated, whereby the focus will be on the development and validation of a new instrument that actively explores suicidal ideation in primary care patients with depression and also explores their reasons for living. Until now, GPs do not have a gold standard instrument to explore patients’ suicidality, although there are some instruments that are used in primary care settings [70].

#### Therapeutic projects (P4-P6)

In the treatment of multimorbidity in primary care, greater patient involvement and coordinated long-term care concepts are necessary to counteract existing uncoordinated and fragmented care [71]. In focusing on "Treatment Challenges", POKAL will seek new approaches to depression treatment by developing and testing structured patient participation and coordinated long-term support based on the CCM. Projects in this area will investigate treatment approaches that are intended to ensure lasting effects and safety, especially in the treatment of depression in patients with multimorbidity.. An important element of CCM is patient monitoring, which is conventionally carried out by a so-called case manager, a specially trained medical or non-medical individual who regularly contacts the patient, coordinates treatment, and monitors, documents, and communicates the patient's progress to the primary care physician. The primary care physician thus receives a regular update on the patient's current health status and can respond and adjust treatment if necessary [72, 73]. Psychoeducation (which main elements are the provision of information, emotional relief, exchange of experiences and support for treatment, e.g. [74]), monitoring, and guidance on pharmacotherapy will be developed for use in family practice and used as part of a comprehensive and synergistic approach that will have an effect on the long-term prognosis of patients with depression.

#### Implementation projects (P7-P9)

Besides diagnostic and treatment, implementation is the third major research field in the POKAL programme.

The translation of innovations in health care services into primary care practice is often difficult. However, simply disseminating knowledge (e.g. in the form of guidelines) is rarely sufficient to ensure it is taken into consideration in routine care. Effective strategies to strengthen evidence-based medicine are needed in somatic as well as in mental health [75]. By analysing the barriers, challenges, incentives ets. and developing adequate implementation strategies in an early stage of research, we hope to optimise translation of our research results into practice.

Related projects will investigate conditions and strategies for the optimized implementation of the instruments developed in the other POKAL projects. New approaches to the collection and utilization of data, guideline use and other contextual factors relating to health care, that have hitherto barely been taken into account, will support the reliable implementation of the newly developed approaches to diagnostics and treatment.

### Collaboration

All POKAL members, PIs and students will participate in consortium task forces covering the following areas: data, doctoral supervision/ombudsperson, media, public relations, gender equality, patient perspectives, networking with GP practices, qualification concepts, strategic development and networking.

The PIs Peter Falkai and Jochen Gensichen have close links to the Else Kröner-Fresenius Research School for "Translational Psychiatry" and the LMU Leadership Program “General Practitioner 360°”, both of which will support and complement POKAL’s future GP training programs. On an international level, collaborations are planned with the co-operation of the advisory board, two of whose board members (Jürgen Unützer, Chris van Weel) have initiated similar programs in the past. (Advancing Integrated Mental Health Solutions (AIMS) Center, http://aims.uw.edu; Netherlands School of Public Health and Care Research https://www.researchschoolcare.nl; Brisbane Initiative on advanced research training for primary care). The Advisory Board of POKAL consists of Jürgen Unützer, University of Washington (USA), Chris van Weel, Raboud University (NL), Brenda Penninx, UMC Amsterdam (NL), Wolfgang Ritter, Bavarian General Practitioners’ Association (D), and Karolina De Valerio (Münchner Bündnis gegen Depression e.V.). Our expertise as well as the continuous input of our (inter)national partners and advisors will promote and maintain POKAL’s high-level of scientific integrity. [76]

## Discussion

This paper summarizes the background and objectives of a German research training school on mental health (POKAL) and of the projects that will be initiated under its umbrella. POKAL gives considerable importance to the training of young (mental) health scientist, providing a high-quality programme and structures to support them. Over the next three years (2021–2024), the first cohort of POKAL trainees will lay the foundation for further research within the consortium.

The underlying theoretical and management concept is based on the chronic care model (CCM), a collaborative care model. Besides improving patients´ health, the use of collaborative care models results in fewer hospitalizations and visits to the emergency room, and improvements in overall care [77–79]. Literature shows the predominantly positive economic impact of the implementation of two or more CCM elements [78]. Evaluating the impact of combining multiple CCM elements will necessitate facing the challenge of disentangling the contribution of individual intervention components [42, 43]. Overall, the implementation of multiple CCM elements appears to be associated with better quality of care [43].

The biopsychosocial model suggests, that biological, psychological and social factors all end up influencing each other and feeding into each other in an interdependent way. Depression can be caused by any number of factors that would on their surface appear to be independent from one another. Thus, the biopsychosocial model encourages clinicians to explain depression by examining all relevant biological, psychological, and social factors that might be contributing to the development or maintenance of the disorder [80, 81]. However, research indicates that psychological problems which are frequently seen and managed in primary care are mostly classified within a few diagnostic categories. GPs focus primarily on biomedical aspects, whereas social factors were rarely considered a contributing factor [82]. *Perspective.*

Based on the results from the starting cohort we plan two trials in the second cohort of projects (2024–2027): One on improved depression diagnostics and one on improved depression treatment. Projects in the second cohort will aim to develop a new overall algorithm for GP depression diagnosis (P1,P2), an intervention for suicide prevention in primary care (P3), a new approach to depression treatment in the general practice with a special focus on psychoeducation and interdisciplinary monitoring (P4,P5), online intervention for patients and GPs to strengthen guideline adherence (P7); to identify contextual health care factors for depression care (P9); to evaluate an intervention to deprescribe antidepressants (P6), a digital data prototype based on patient-collected data (P8).

### Limitations

Effective treatment of depression requires the combination of pharmacological and psychotherapeutic interventions, and demands a theoretical paradigm integrating biological and psychosocial aspects of depression. However, in some projects we have to limit ourselves to a narrower, categorical understanding of depression for reasons of practicability, at least in this early phase. At this stage, projects in POKAL do not investigate precursors to depression/the prodromal phase of depression. We investigate depression and its comorbidities. We also see the potential of transdiagnostic research in POKAL: transdiagnostic approaches are becoming more and more important in primary care; although we are currently looking at depression specifically, we might involve transdiagnostic research questions in a later stage of POKAL.

## Conclusion

The research training group POKAL aims to provide appropriate approaches for depression diagnosis and treatment in primary care. POKAL strives to generate well-trained GPs and other health care professionals who will later support the health care system with their expertise.

## Data Availability

Not applicable. The manuscript does not contain any data, because it describes the conceptual framework of a research training group (POKAL) funded by the German Research Foundation (DFG—GrK 2621 / POKAL-Kolleg).
